# Exhaled carbon monoxide as a marker of lung aging in smokers presenting with mild air flow limitation

**DOI:** 10.1186/1617-9625-12-S1-A9

**Published:** 2014-06-06

**Authors:** Marios Kougias, Maria Takousi, Aristea Baschali, Christina Goga, Nikolaos Tatsis, Stavroula Boulia, Evangelos Balis, George Tatsis

**Affiliations:** 1 Smoking Cessation Department, Pulmonology Clinic, Evaggelismos General Hospital, Athens, 10676, Greece

## Background

The measurement of exhaled carbon monoxide (CO) level may provide an immediate, noninvasive method of assessing smoking status. In addition CO is a molecule generally presumed to be a marker of oxidative stress and inflammation when endogenously produced. Many reports have focused on increased endogenous CO production in pulmonary diseases, including asthma and COPD. The purpose of our study is to highlight special characteristics in smokers with mild airflow limitation, in correlation with exhaled CO measurements.

## Materials and methods

The exhaled CO levels were measured in 29 smokers (18 women),of mean age 50.62 ± 9.55 smoking 43.28 ± 29.50 pack years presenting at the smoking cessation office of Evaggelismos Hospital. A thorough smoking history was obtained focusing on smoking use, dependence and motivation to quit. Exhaled CO measurement was performed using the piCO+™ Smokerlyzer (Bedfont Instruments; Kent, UK), which also allows the accurate estimation of the percentage of hemoglobin attached to CO(HBCO). Spirometry was performed in all individuals according to ATS criteria, and all participants measured FEV1>80% of predicted. Data were analyzed using descriptive statistics and Spearman correlation analysis.

## Results

FEV1 measured 89.28% ± 3.84 FVC measured 94.63% ± 3.55 and MMEF measured 65.02% ± 5.79. CO measured 25.48ppm ± 2.90. HBCO was estimated at 4.71% ± 0.45. Results are expressed as mean ±SE. Exhaled CO correlated with age of measured subjects (correlation coefficient -0.379 at p=0.01 double-sided). HBCO also correlated with age (correlation coefficient -0.387 at p=0.01 double-sided).

## Conclusions

Exhaled CO is a noninvasive method of smoking status but is also a marker of oxidative stress and inflammation .Correlation of CO measurement with the age of smokers may provide an insight in the inflammatory process of smoking, in smokers not presenting with severe airflow limitation, and provide a marker of lung aging in smokers. Exhaled CO besides a method for assessing smoking status may provide useful information of lung aging due to lung inflammation, in smokers with mild airflow limitation.

